# A Rare Case of Bertolotti's Syndrome in a Young Patient: A Case Report and Literature Review

**DOI:** 10.7759/cureus.10957

**Published:** 2020-10-15

**Authors:** Jasvindar Kumar, Sundas Ali, Nasrullah Zadran, Manjeet Singh, Zahoor Ahmed

**Affiliations:** 1 Internal Medicine, Khyber Teaching Hospital, Peshawar, PAK; 2 Internal Medicine, Holy Family Hospital/Rawalpindi Medical University, Rawalpindi, PAK; 3 Internal Medicine, Lady Reading Hospital MTI, Peshawar, PAK; 4 Pathology, Liaquat National Medical College, Karachi, PAK; 5 Pathology, Liaquat National Hospital, Karachi, PAK; 6 Internal Medicine, King Edward Medical University, Mayo Hospital, Lahore, PAK

**Keywords:** bertolotti's syndrome, back pain, lumbar vertebra, treatment, young patient

## Abstract

Bertolotti's syndrome is a congenital condition characterized by the sacralization of the lower lumbar vertebrae or the lumbarization of the sacral vertebrae. The cause of pain in Bertolotti’s syndrome is multifactorial. This lumbosacral transitional vertebra has a prevalence of 4% to 30%. Rarely, it is considered in the differential diagnosis of low back pain in young people. Therefore, every aspect of Bertolotti’s syndrome needs to be meticulously addressed, and it should be included in the differential diagnosis of chronic back pain. Herein, we present a case of Bertolotti’s syndrome presented with chronic lower back pain, confirmed on X-ray and magnetic resonance imaging (MRI). He was managed with analgesics and steroids injection with regular follow-up.

## Introduction

Bertolotti's syndrome (BS), or lumbosacral transitional vertebrae, is the partial or complete fusion of the unilateral or bilateral most caudal lumbar vertebrae to the sacrum or ilium. It is an anomalous enlargement of the transverse process of the fifth lumbar vertebrae that either fuses or articulates with the sacrum or ilium bone and causes L4-L5 disc disease. This relationship was first studied by Bertolotti in 1917, who relates this to low back pain. This anatomical variation may lead to a change in the biomechanics of the movement of the spine and may cause lower back pain [1]. Initially, it was noted that anatomic variation affects 4% to 8% of the population but a higher incidence of 30% was noticed [1-2]. Herein, we present a rare case of BS in a young 20-year-old patient who presented with chronic low back pain.

## Case presentation

**Figure 1:**
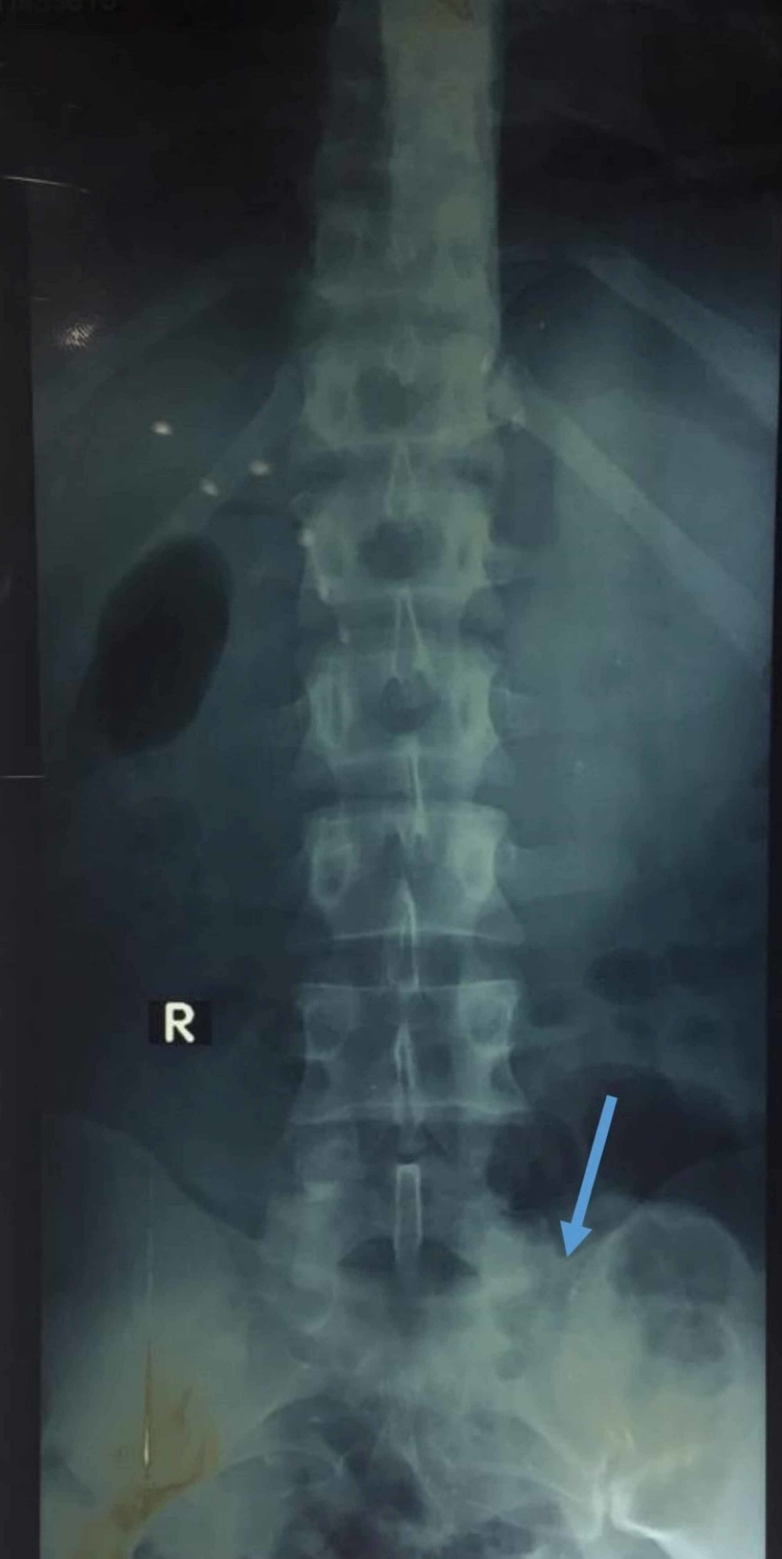
Anteroposterior pelvic radiograph showing sacralization of the lumbar vertebrae (blue arrow)

 A 20-year-old male with no previous history of trauma presented with lower back pain. The pain was dull, non-radiating, and localized to the lower back. The backache started one year back when the patient exercised excessively and lifted some heavyweights. Initially, it was considered as muscle sprain, and his physician prescribed analgesics and advised him to take rest. He experienced pain often, which was relieved by pain killers. On initial evaluation, his temperature was 37°C, blood pressure of 120/70 mmHg, heart rate of 91 beats per minute, respiratory rate of 20/minute, oxygen saturation of 99% on room air. On physical examination, there was no redness, swelling, tenderness, and warmth. On movement of the spine, he experienced pain on the bending in the lower back, and limitations to full rotation were also noticed. The straight leg raised test was negative. The patient has no radiation to lower limbs. The reflexes, sensations, and lower limb power was intact. Radiographic studies show an enlarged transverse process of the lower lumbar vertebrae, which fuses with the sacrum (Figure 1).

No arthritic changes were noticed. Magnetic resonance imaging (MRI) was performed, which revealed a fusion of the transverse process of lower lumbar vertebrae with the sacrum and confirmed a diagnosis of Bertolotti’s syndrome. The patient was put on vitamin D, calcium supplements, and some analgesia. However, he did not experience any improvements to his symptoms. He was commenced on analgesics with steroids injection. Home physiotherapy was also advised, which included stabilizing and strengthening exercises for the lower lumbar spine. On follow-up three weeks later, he was doing well with no active complaint.

## Discussion

Lumbosacral transitional vertebrae is a common variant and seen in 25% (range 15% to 35 %) of the general population. Quinlan et al. claimed that BS has a considerable influence on young patients, with the total incidence being 4.6% and a frequency of 11.4 % in the under 30-year age group. They can be the lumbarized S1 segment or sacralized L5 segment. A varying degree of partial to complete fusion transition has been noticed. This transition can be unilateral or bilateral, which leads to back pain in the second decade of life in most patients. Mechanical and biomechanics changes occur that affect the facet joints; overstrain of the psoas and quadrate lumborum muscle and stenosis of the intervertebral foramen may compress nerve roots and the extrusion and protrusion of the disc also cause lumbosciatic pain in some patients.

The pain in BS is attributed to multiple factors. It could be because of facet joint arthropathy (scoliosis), muscle strain (iliopsoas and quadratus lumborum) [3]. The pseudoarthrosis or asymmetry between transitional vertebrae and sacrum joint leads to hypermobility and early degeneration of the proximal joint, which, in turn, is subjected to increased stress and limited vertebral mobility [1]. Whether transitional vertebrae produce low back pain or not is a matter of debate [4]. Otani et al. reported a higher incidence of the lumbosacral joint in patients with disc herniation (17%) than in the control group(11%) [5]. The disc between the transitional vertebra and sacrum is comparatively less degenerative as compared to the disc above it, which is prone to more significant degenerative changes (disc protrusion or extrusion). Similar effects were observed in the proximal joint with nerve canal stenosis. The transitional joint does not cause nerve root symptoms, which might be considered a risk factor for nerve root compression in patients with lumbar canal stenosis without spondylolisthesis or disc herniation [5].

The diagnosis is based on the detailed history and the radiographic changes that appeared at the surrounding structures of the lumbosacral transitional vertebra [6]. It has been reported that the facetogenic joint reduces the annulus fibrosis degeneration of the disc [4]. Scoliosis is the most consistent feature in X-ray imaging. Proximal joint degeneration has been identified in association with disc herniation in the transitional lumbar vertebra, and MRI assists in the diagnosis of intervertebral disc protrusion [7-8]. Diagnosis missed by plain X-ray could be identified by bone scan, computed tomography of the pelvis, or single-photon emission computed tomography, which further helps in detailing bone structure, osteophytes, and pseudo articulation of the transverse process. Increased uptake on bone scan seems promising in identifying the source of back pain and helps in the assessment of the origin of pain from the facet joint or sacroiliitis [9].

In 1984, Castellvi et al. described a radiographic classification system identifying four types of lumbosacral transitional vertebrae (LSTVs) based on morphologic characteristics. Type I include unilateral (Ia) or bilateral (Ib) dysplastic transverse processes measuring at least 19 mm in width in the craniocaudal dimension. Type II LSTV exhibits incomplete unilateral (IIa) or bilateral (IIb) lumbarization/sacralization with an enlarged transverse process that has a diarthrodial joint between itself and the sacrum type III LSTV describes unilateral (IIIa) or bilateral (IIIb) lumbarization/sacralization with complete fusion of osseous of the transverse process to the sacrum. Type IV involves a unilateral type II transition with a type III on the contralateral side. As seen in the literature, LSTV type II to IV was related to BS while type I remained asymptomatic, and most cases are found accidentally. Castellvi et al. also state the prevalence of the transitional joint in patients presenting with sciatica and back pain to be 30% and 21%, respectively [10]. To date, there is no consensus on the best method of treatment for BS patients. The therapeutic approach of BS is conservative, pharmacological, and surgical. Corticosteroid and local anesthesia is considered the first-line agent for the treatment of low back pain and was reported to resolve backache when administered in the cavity of pseudoarthrosis. It also expands the transverse process of the transitional vertebra, which further resolves the symptoms. Nonsteroidal anti-inflammatory drugs, corticosteroids, and muscle relaxants, especially corticosteroids and local anesthetics, can be injected into articulation for both diagnostic and treatment purposes. If conservative treatment is unsuccessful, surgery can be considered, which includes the resection of the transverse process if no disc pathology is suspected. A study by Santavirta et al. revealed that 60% of patients showed improvements in the surgical resection of the transverse process. In case of discogenic pain, posterolateral fusion, blockage of afferent rami communicants from a paravertebral sympathetic chain or the white ramus of L1 and L2 at a vertebral level adjacent to the painful disc has proved helpful in elucidating pain [9].

## Conclusions

Chronic low back pain in young patients could be the presenting sign of Bertolotti’s syndrome though it is highly uncommon. Due to its rarity and variable clinical presentations, a high index of clinical suspicion is required for the diagnosis of Bertolotti’s syndrome. Therefore, it should be included in the differential diagnosis of chronic low back pain after ruling out all other possible causes. Clinicians should have sound knowledge and pay close attention to chronic low back pain and its possible causes.

## References

[1] Luoma K, Vehmas T, Raininko R, Luukkonen R, Riihimäki H (2004). Lumbosacral transitional vertebra: relation to disc degeneration and low back pain. Spine.

[2] Aihara T, Takahashi K, Ogasawara A, Itadera E, Ono Y, Moriya H (2005). Intervertebral disc degeneration associated with lumbosacral transitional vertebrae: a clinical and anatomical study. J Bone Joint Surg Br.

[3] Quinlan J, Duke D, Eustace S (2006). Bertolotti’s syndrome: a cause of back pain in young people. J Bone Joint Surg Br.

[4] Bonaiuti D, Faccenda I, Flores A (1997). Sacralization of the 5th lumbar vertebra and backache: what's the possible relationship? [Article in Italian]. Med Lav.

[5] Otani K, Konno S, Kikuchi S (2001). Lumbosacral transitional vertebrae and nerve-root symptoms. J Bone Joint Surg Br.

[6] Southwood J, Bersack S (1950). Anomalies of the lumbosacral junction in 550 patients without symptoms referable to the low back. Am J Roentgenol Radium Ther.

[7] Vergauwen S, Parizel PM, van Breusegem L, Nackaerts Y, Van den Hauwe L, De Schepper AM (1997). Distribution and incidence of degenerative spine changes in patients with a lumbo-sacral transitional vertebra. Eur Spine J.

[8] Magora A, Schwartz A (1978). Relation between the low back pain syndrome and x-ray findings. 2. Transitional vertebra (mainly sacralization). Scand J Rehabil Med.

[9] Jain A, Agarwal A, Jain S (2013). Bertolotti syndrome: a diagnostic and management dilemma for pain physicians. Korean J Pain.

[10] Castellvi AE, Goldstein LA, Chan D (1984). Lumbosacral transitional vertebrae and their relationship with lumbar extradural defects. Spine.

